# The Associations Between Mental Health Problems and Attitudes Toward Web-Based Health and Social Care Services: Evidence From a Finnish Population-Based Study

**DOI:** 10.2196/28066

**Published:** 2021-09-21

**Authors:** Teemu Rantanen, Kia Gluschkoff, Piia Silvennoinen, Tarja Heponiemi

**Affiliations:** 1 Unit of Digital Education and Master Programmes Laurea University of Applied Sciences Vantaa Finland; 2 Welfare State Research and Reform Finnish Institute for Health and Welfare Helsinki Finland; 3 Department of Psychology and Logopedics University of Helsinki Helsinki Finland

**Keywords:** digital inclusion, digital exclusion, digital divide, mental health, attitudes

## Abstract

**Background:**

The significance of web-based health and social care services has been highlighted in recent years. There is a risk that the digitalization of public services will reinforce the digital and social exclusion of vulnerable groups, such as individuals with mental health problems.

**Objective:**

This study aims to examine the associations between mental health problems and attitudes toward web-based health and social care services in the general population. The attitudes measured include lack of interest, perceived need for face-to-face encounters, and concern for safety. The study also evaluates whether sociodemographic characteristics (age, gender, education level, and poverty) modify these associations.

**Methods:**

Cross-sectional population-based data were collected from 4495 Finnish adults in 2017. Linear regression was used to examine the main effects and interactions of poor mental health and sociodemographic characteristics on attitudes toward web-based health and social care services.

**Results:**

The results show that mental health was associated with attitudes toward web-based health and social care services. Individuals with mental health problems were especially concerned about the safety of web-based services. Poor mental health was independently associated with negative attitudes toward web-based services over the effects of sociodemographic factors. Some of the associations between poor mental health and negative attitudes toward web-based services were stronger among older people and men. With regard to sociodemographic characteristics, particularly higher age, low education, and poverty were associated with negative attitudes toward web-based health and social care services.

**Conclusions:**

Poor mental health is associated with negative attitudes toward web-based health and social care services and thus indirectly with exclusion. It seems that being older and being male both reinforce the link between poor mental health and exclusion. In supporting the digital inclusion of people with mental health problems, attention should be paid to guidance and counseling, reliability, and the user-friendliness of web-based services as well as to the prevention of poverty. In addition, it is essential to see web-based services as complementary to, and not a substitute for, face-to-face services.

## Introduction

### Background

The push for web-based health and social care services is increasing at an accelerating pace in developed countries. This development is based on the assumption that patients and clients are able to independently engage with services and experience them as beneficial [1-4]. However, there is an increasing number of vulnerable groups whose capability to engage with services is poor. In addition, some individuals either do not engage at all or choose to disengage from web-based services [2-4]. Thus, the digitalization of health and social care services might lead to a greater digital divide and more exclusion than anticipated because of the perceived burden associated with web-based services among vulnerable groups [2,3].

Various vulnerable groups of people, such as those with mental health disorders, are at risk of digital exclusion. According to Greer et al [5], mental health difficulties, specifically psychosis, impact digital exclusion. For example, a lack of knowledge and deficiencies in financial resources can be significant risk factors for digital exclusion among mental health service users [5]. Farooq et al [6] argued that digital inclusion cannot be considered separately from economic and social inclusion. According to them, it is likely that socially disadvantaged and mentally ill people will lag behind the rest of the population if digital inclusion is not addressed as a priority issue. In addition, other factors such as education level, age, and social deprivation play an important role in estimating who are willing and able to use digital services [7,8].

Attitudes can be seen as one component of digital inclusion [9,10]. According to the Helsper [11] theory, social exclusion increases the risk of digital exclusion via a lack of digital skills, poor access to the internet and digital services, and negative attitudes toward technology. The central role of attitudes has also been highlighted in research on the adoption of new technologies [12]. According to the technology acceptance model [13] and the theory of planned behavior [14], attitudes are one of the key factors in the adoption of and behavioral intention to use technology. Therefore, people’s attitudes affect how willing they are to use new technology.

The concept of attitude is multidimensional and difficult to define; however, attitude is typically understood as the valuation of a given target [15]. As people with mental health problems have been found to be critical of face-to-face services being replaced by digital services [16,17], this study focuses first on the ways in which people view digital services compared with face-to-face services. As previous studies have shown that a user’s belief in a specific service posing no security or privacy threats is an important factor in the adoption of a web-based service [18,19], this study also examines the perceived trustworthiness of web-based health and social care services. According to Gong et al [20], trust in the provider also affects the perceived benefits and risks of web-based services. Furthermore, the technology acceptance model posits that perceived usefulness is an important factor in the acceptance of new technology [13]. Therefore, as an additional dimension, this study examines attitudes regarding the perceived usefulness of web-based services and the general interest in using them.

In summary, this study approaches the issues of digital inclusion and exclusion from the perspective of attitudes. Specifically, we studied the combined effects of poor mental health and sociodemographic characteristics on attitudes toward web-based health and social care services.

### Mental Health, Sociodemographics, and Web-Based Services

Although aggregate digital inclusion has improved overall, the inclusion gap has widened in recent years [10]. Moreover, it has been suggested that the digital exclusion of vulnerable groups will increase rather than decrease in the future [2,3]. The degree of digital literacy and people’s ability to use digital services vary within age groups and align with socioeconomic status [2,3]. The greater an individual’s offline resources, the more information and communication technology leads to beneficial economic, social, and educational outcomes [2,3,21]. There are several key factors, such as use, income, and education, that discriminate between web-based and offline health information seekers. Indeed, individuals who seek health materials on the web, for example, are likely to be younger, have a better socioeconomic position, and themselves feel happier than those who report only seeking health information offline [22].

A study by Chen and Zhu [8] concluded that youth and high socioeconomic positions are significant predictors of internet access and use. Previous findings also show that older individuals with mental health disorders tend to have fewer skills and financial resources and less familiarity, confidence, and access in engaging with and using digital mental health services than their younger counterparts [23,24]. Moreover, it has been shown that older individuals with mental health disorders do not experience these services as beneficial and do not wish to engage with technology [23,24].

In a Finnish study [25], 89% (40/45) of adolescents with symptoms of depression or anxiety who used a web-based depression support system experienced the support system as reliable and safe. Specifically, the support system was experienced as safe and beneficial when users were able to trust the system’s content and felt safe using it. In addition, the criteria for reliability and safety were met when web-based mental health treatment was carried out in a way that protected the confidentiality of adolescent users. A further study on people living with serious mental health illnesses highlights the importance of social media in conducting symptom assessment, as well as in peer support [26].

People belonging to vulnerable groups are more likely to be cynical toward web-based health and social care services and to prefer face-to-face interaction over web-based interactions [16,17,27-29]. The communication technology involved is considered to not convey warmth, empathy, and nonverbal cues at the same depth as direct human contact [16,17,30]. In addition, vulnerable groups have been found to be suspicious of the confidentiality, safety, and privacy of web-based health and social care services [16,17,27-29]. Furthermore, a systematic review of the acceptability of web-based- and mobile phone–delivered interventions indicated that people with severe mental health problems are generally reluctant to engage with these interventions [28].

A study exploring the reception and use of computerized cognitive behavioral therapy in the treatment of depression among university students concluded that a significant proportion of the participants preferred face-to-face contact alongside computer-assisted therapy [27]. In general, participants also experienced computerized treatment as less credible than face-to-face counseling, and attempting to use a computerized cognitive behavioral therapy program alone might be too laborious a task to undertake when depressed [27]. Similarly, a further qualitative study involving those with severe mental health problems highlighted that participants experienced digital health interventions as merely a complementary method to supplement face-to-face care [17]. Digital health interventions were not perceived as helpful and effective as face-to-face interactions, and some individuals were concerned about the confidentiality of digital services. A Finnish study [16] found that marginalized young people prefer face-to-face interaction over web-based interaction when they want to discuss their problems with a social worker, despite being quite fluent in the use of digital devices for personal purposes. However, older adults have been found to lack confidence and trust in sharing personal information and tend to be suspicious of the ways in which the collected data are processed and their privacy maintained [29].

In addition, previous studies have identified a digital gender gap, for example, in digital skills, and access to and the use of digital services and technologies [31-33]. However, according to Thomas et al [9], gender is not as significant a risk factor for low digital inclusion as, for example, low income, a low level of education, or older age. Attitudes, on the other hand, are the dimensions of digital inclusion in which the gender gap is greatest. Therefore, it is justified to also look at the gender gap when examining digital attitudes.

In general, women have been found to be more likely to search for health-related information on the web than men, but men are more open to engaging in a virtual relationship [34]. Furthermore, previous studies have found significant differences in the help-seeking behaviors of men and women with mental health problems. In fact, males, young people, and people living in affluent areas, for example, were the least likely to seek help [35]. Moreover, according to Smail-Crevier et al [36], women were found to be more likely to use the internet for medical or health-related information and guidance on reducing symptoms of stress and depression, as well as an interactive self-help software program, whereas men preferred to receive information in a video game format.

Women have been found to perceive cognitive behavioral therapy delivered on the web as more acceptable and easier to adhere to than men [37]. However, according to Zhang et al [38], men have a higher level of intention toward mobile health adoption than women. Accordingly, Ellis et al [39] pointed out that the key challenge for web-based mental health services is to design interventions specifically for young men that are action based, focus on shifting behavior and stigma, and are not simply about increasing mental health knowledge. However, not all studies have found gender differences. For example, in the Australian study of Klein and Cook [40], the majority of respondents stated a preference for using traditional mental health services, and no differences were found in relation to any demographic variable or previous use of mental health services between groups of e-preferers and non-e-preferers [41].

### Aims

First, previous studies have shown that attitudes are significant factors of influence in the acceptance and use of new technologies [12-14] and in digital exclusion [9,11]. Second, studies have also shown a link between mental health problems and negative attitudes toward web-based services [16,17]. Finally, previous studies suggest that sociodemographic factors influence the use of web-based services [7,8]. However, there is a lack of research on the common effects of mental health problems and sociodemographic factors on attitudes toward web-based services. Moreover, although previous research has examined the moderating effects of socioeconomic characteristics on the association between mental health and health services use [42], web-based services have not received attention in the relevant empirical literature.

This study examined the attitudes toward web-based health and social care services. The measures of attitudes used included *lack of interest*, *perceived need for face-to-face encounters*, and *concern for safety*. In particular, the study analyzed the effects of poor mental health and sociodemographic characteristics (age, gender, education level, and poverty) on these attitudes. Moreover, the moderating effect of sociodemographics on the association between mental health problems and attitudes was examined.

## Methods

### Sample and Procedure

This cross-sectional study was based on a random sample representative of the Finnish population. In Finland, although almost all people aged <45 years use the internet every day, this use decreases with age. For example, 90% of people aged 45-54 years use the internet every day, compared with 83% of people aged 55-64 years, and only 57% of people aged 65-74 years use the internet every day [43]. The majority of Finns (80%) use mobile phones to access the internet [43]. Although the use of the internet and the skills required to do so are generally common in Finland, there are gaps in digital skills and an obvious need for support among some socially marginalized groups [44].

A sample of 10,000 people was collected from the Population Register Center of Finland. For those aged ≥75 years, a double-picking probability was used to guarantee a sufficient group size. A questionnaire was sent by mail to the participants in 2017. The paper questionnaire form also provided instructions for answering the questionnaire on the web, if preferred. Reminders were sent three times to those who did not respond.

The material is collected by post using a paper form or via a web-based form. Only a very limited number of people have access to a logistics system in which forms are managed. A secure connection is used for the web-based form, and the answers are stored encrypted in the Finnish Institute for Health and Welfare (THL) database. In the analysis phase, the data obtained from paper forms and electronic forms were combined in a research database. The analyses used pseudonymized research data from which direct personal data were removed. The identification information is stored on the THL’s network drive, which can only be accessed by THL employees responsible for managing the material.

The total number of respondents was 4495, yielding a response rate of 44.95% (4495/10,000). The respondents were slightly older, more often women, and had a higher education level than the eligible general population [45]. Inverse probability weighting was applied to address possible bias based on age, gender, marital status, education level, living region, and the degree of urbanization of the residential municipality. This method has previously performed well in correcting the possible effects of nonresponse on the representativeness of the results [46]. Owing to missing values in some of the study variables (Table 1), the analytic sample size ranged from 3200 to 3459, depending on the analysis being carried out.

**Table 1:** Descriptive statistics (N=4495).

Variable			Value
Age (years), mean (SD)			51.34 (18.38)
**Gender, n (%)**			4495 (100)
	Female	2301 (51.19)	
	Male	2194 (48.81)	
**Education, n (%)**			4259 (94.75)
	High	1328 (31.18)	
	Average	1440 (33.81)	
	Low	1491 (35.01)	
**Poverty, n (%)**			4344 (96.64)
	Yes	807 (18.58)	
	No	3537 (81.42)	
**Poor mental health, n (%)**			3853 (85.72)
	Yes	569 (14.76)	
	No	3284 (85.24)	
**Attitudes (scale 1-5), mean (SD)**			3643 (81.04)
	Lack of interest	2.13 (1.27)	
	Need for face-to-face encounters	3.16 (1.13)	
	Concern for safety	2.62 (1.25)	

The study complied with the instructions provided by the Finnish National Board on Research Integrity [47] and the ethical principles of the World Medical Association Declaration of Helsinki [48]. Ethical approval for the study was obtained from the Research Ethics Committee of THL (THL/637/6.02.01/2017).

### Measures

#### Attitudes Toward Web-Based Health and Social Care Services

Although the attitude questions on the form were not directly based on any particular theoretical approach, the questions concerned different aspects of the attitudes toward web-based services identified in previous studies [12,16-20]. When preparing the form, attitude questions were considered from the perspective of learning and quality and, for example, in relation to the information systems success model [49], which emphasizes the importance of information quality, system quality, service quality, user satisfaction, and net benefits for use. In addition, the questions used in the citizens' surveys of other countries were taken into account to allow comparisons between countries.

The respondents were asked, “What factors make it impossible or difficult for you to use online social and health care services?” The response options (rated on a scale from 1=completely disagree to 5=completely agree) measured three types of negative attitudes toward web-based services: *lack of interest* (“Electronic services do not interest me;” Cronbach α=.92), *perceived need for face-to-face encounters* (*“*Face-to-face encounters cannot be replaced by electronic contacts;” Cronbach α=.81), and *concern for safety* (“I am concerned about data security when it comes to my personal details;” Cronbach α=.91). These three dimensions of limiting attitudes were derived using factor analysis (see Table S1 of Multimedia Appendix 1 [50,51]).

#### Poor Mental Health

Poor mental health was operationalized using both (1) the Mental Health Inventory (MHI)-5 and (2) responses to a single question enquiring about recent treatment for or diagnosis of depression (“Have you been diagnosed with depression or been treated for depression by a doctor over the past 12 months?”). The MHI-5 [52] is a short 5-item scale that measures feelings of depression and anxiety. More specifically, the MHI-5 measures mental health in the domains of anxiety, general positive affect, depression, and behavioral or emotional control. Respondents who scored ≤52 points on the MHI-5 or indicated that they had been recently diagnosed or treated for depression were classified as having poor mental health.

#### Sociodemographic Characteristics

Sociodemographic characteristics included age (continuous variable), gender, level of education, and poverty. Education level was measured with the years of education (top coded at 30 years and divided into age-group specific [<26, 26-35, 36-45, 46-55, ≥56 years] tertiles; 1=high, 2=average, and 3=low). Poverty was measured with three items enquiring about not having money for food, medication, or medical treatment. (“Have you within the past 12 months ever feared that you will run out of food before you can get money to buy more”). Responding *yes* to any of the three items was used as an indicator for poverty.

### Statistical Analyses

Linear regression analysis was used to examine the associations. Poor mental health and sociodemographic factors (age, gender, education, and poverty) were the independent variables, and the factor scores of the three factors for limiting attitudes were the dependent variables. Each attitude was examined using a separate regression model. The analyses were conducted in three steps for each of the three outcome variables. First, unadjusted analyses were performed to examine the associations between poor mental health and negative attitudes (step 1). Second, we adjusted the analyses for the effects of age, gender, education, and poverty (step 2). Quadratic age was additionally included in the analyses to test for potential nonlinear associations between age and attitudes. Finally, the interactions between poor mental health and sociodemographic characteristics were tested (step 3). All analyses were conducted using sampling weights in R version 3.6.1 (R Foundation for Statistical Computing) [53].

## Results

### Main Effect Models

Descriptive statistics for the sample are shown in Table 1, and bivariate associations are shown in Table S2 and Figure S1 of Multimedia Appendix 1. On average, the sample was 51.3 (SD 18.38) years, and approximately half were female (2301/4495, 51.19%). The results of the regression analysis (steps 1 and 2) are presented in Table 2. Poor mental health was associated with negative attitudes toward web-based health and social care services, with regard to all three types of attitudes (step 1). The association between poor mental health and negative attitudes was strongest in terms of *concern for safety*. The associations were attenuated but remained significant after adjusting for sociodemographic factors (step 2). In terms of sociodemographic factors, higher age and lower education were associated with all types of negative attitudes. Some of the associations of age were nonlinear, with stronger associations among older respondents. Being a woman and experiencing poverty were (weakly) associated with the *need for face-to-face encounters* and *concerns for safety*.

**Table 2:** Results of regression analysis.

Predictor				Attitudes												
				Lack of interest					Need for face-to-face encounters					Concern for safety		
				Value		*P* value		Value			*P* value		Value			*P* value
**Step 1 (n=3459)**																
	Poor mental health, β		.24		*<.001* ^a^		.34			*<.001*		.38			*<.001*	
	R^2^ (%)		1		N/A^b^		2			N/A		2			N/A	
**Step 2 (n=3200), β**																
	Poor mental health		.20		*<.001*		.28			*<.001*		.31			*<.001*	
	Age		.33		*<.001*		.31			*<.001*		.29			*<.001*	
	Age^2^		.13		*<.001*		.06			*<.001*		.03			.10	
	Gender (female)		.05		.13		.09			*.008*		.07			*.047*	
	**Education, β**															
		High	Reference		N/A		Reference			N/A		Reference			N/A	
		Average	.17		*<.001*		0.11			*<.001*		.17			*<.001*	
		Low	.42		*<.001*		0.25			*.008*		.30			*<.001*	
	Poverty (yes), β		.06		.15		0.19			*<.001*		.19			*<.001*	
	R^2^ (%)		23		N/A		14			N/A		13			N/A	

^a^Significant *P* values (*P*<.05) are shown in italics.

^b^N/A: not applicable.

### Interactions

**Figure 1:**
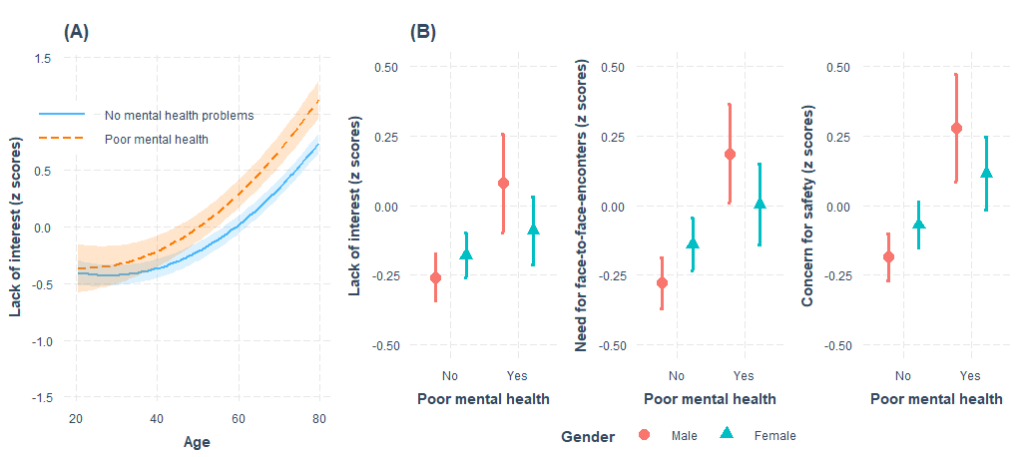
The interaction (A) between poor mental health and age and (B) between poor mental health and gender in predicting negative attitudes toward web-based health and social care services.

Regarding the interactions between poor mental health and sociodemographic factors (step 3), age moderated the association of poor mental health with a lack of interest in web-based services (β=.10; *P*=.03). The association between poor mental health and a lack of interest was stronger among older adults. Gender moderated the association between poor mental health and *lack of interest in web-based services* (β=−.25; *P*=.02), *need for face-to-face encounters* (β=−.32; *P*<.001), and *concerns for safety* (β=−.28; *P*=.01). Compared with women, the association of poor mental health with all types of negative attitudes was stronger among male respondents. These interactions are shown in Figure 1.

## Discussion

### Principal Findings

This study found a significant difference in attitudes toward web-based health and social care services between people with mental problems and others. People with mental health problems were more likely to be concerned about using web-based services and have a lack of interest in them compared with other people. Moreover, they were more likely to consider web-based services as not being able to replace face-to-face encounters. The associations between poor mental health and negative attitudes remained significant, although other factors, such as age, gender, low education level, and poverty, were included in the model. Thus, according to our results, poor mental health is an independent risk factor for negative attitudes toward web-based services. In addition, we found that older age, low education level, and poverty increased the criticality of attitudes. The significance of gender was particularly reflected in the fact that women emphasized the importance of face-to-face encounters more than men.

We found that the effects of low levels of education and poverty were similar among people with mental health problems and among other people. In addition, our study shows that age has a significant effect on attitudes toward web-based health and social care services, both among people with mental health problems and among other people. Moreover, age intensifies the association between poor mental health and attitudes. Among young people, interest in using web-based health and social care services did not depend on mental health conditions. In contrast, older people had more negative attitudes toward web-based services, with poor mental health further increasing this negativity. Furthermore, gender moderated the association of poor mental health with attitudes, and the association of poor mental health with a *lack of interest in web-based services*, the *need for face-to-face encounters*, and *concerns for safety* was stronger among men than among women.

### Reflection on Results

Mental health services are increasingly digital, similar to other social and health services. Various web-based therapies have recently been developed, and public mental health services have also begun to be provided on the internet. In addition, free-to-use websites have been built that provide information, tests, and guidelines related to mental health issues. On the basis of the results of this study, it is reasonable to assume that individuals with poor mental health are at risk of digital exclusion and, thus, also social exclusion. However, they were not cohesive groups. Previous studies have identified three major themes that perpetuate digital exclusion among mental health service users: gaps in knowledge, personal circumstances, such as financial barriers, and poor mental health [5]. Consistent with this, this study also highlights the effects of economic factors and mental health on attitudes toward web-based health and social care services and, thus, indirectly also on digital inclusion. However, personal support has been seen as a key factor in improving the digital inclusion of vulnerable people [5,44].

On the basis of this study, it appears that challenges are particularly acute for individuals with mental health problems who have a lower level of education, are older, or are poorer. Therefore, personal support related to the use of services should be targeted specifically for these groups of people. Overcoming the digital exclusion of people with poor mental health also requires addressing wider societal issues related to finance and living circumstances [5].

There are distinctions between age groups because the younger generations have more positive attitudes toward and trust in web-based health services, for example, in the treatment of mental health disorders [25,54]. In fact, the age-related digital divide is not yet narrowing [10]. It can be assumed that new digital tools will especially reach young people with mental health problems. However, for older people and service users with other risk factors, it is also important that the opportunity for face-to-face service continues in the future and that adequate support for the use of digital services is provided. Otherwise, there is a risk that a significant proportion of clients will drop out of mental health services.

On the other hand, the level of education and socioeconomic status play an important role in the adoption of digital services and use of the internet, even among young people who are labeled as technologically more savvy than older generations [7,55]. According to Robards et al [55], marginalized young people particularly need assistance and guidance to use digitalized health care services.

Previous studies have shown that the use of web-based services is related to gender. Although men’s attitudes toward internet use and technology are generally seen to be more positive than women’s [9], women are more willing to seek help using eHealth services [36,39]. The analyses of this study show that the gender gap is different among people with mental health problems, as well as among other people. This finding is new but not entirely surprising. The general interest in using the internet may also increase men's interest in using general eHealth services. On the other hand, if a person needs to seek help for a mental health problem, for example, the situation is likely to be different because of the stigma associated with the service, among other things. The greater willingness of women to seek professional help [35] can also be a significant factor in the use of eHealth services. However, our findings highlight the need to pay special attention to men with mental health problems when developing eHealth services.

The results show that mental health is related not only to general attitudes but also to more specific attitudes. These results are consistent with those of previous studies. According to previous studies, people with severe mental health problems are reluctant to engage in web-based and mobile phone–delivered interventions [28]. They experience digital health interventions merely as complementary methods to face-to-face care [8]. Correspondingly, a study by Mitchell and Gordon [27] showed that a large proportion of people with depression prefer face-to-face contact alongside computerized cognitive behavior therapy. Granholm [16] has suggested that marginalized young people prefer face-to-face interaction over web-based interaction when they want to discuss their problems with a social worker, even if they are otherwise competent in the use of digital devices. In addition, people with mental health problems have been shown to be concerned about the confidentiality of digital services, having doubts about issues of data protection and handling [17]. This study found critical attitudes toward replacing face-to-face interactions with web-based interactions among people with mental health problems. Similarly, this study supports the view that vulnerable groups are suspicious of the confidentiality, safety, and privacy of web-based health and social care services. In addition to providing face-to-face support and guidance, issues related to information security and the reliability of web-based services are seen as key to encouraging people with mental health problems to use web-based services.

It is important to improve the usability of these services to promote positive attitudes toward web-based services. This would be especially important for vulnerable groups, such as those with mental health problems and older people. Focusing on better technical solutions, usability, and user needs when designing solutions has been found to lead to more positive user experiences [56]. Moreover, improving the usability of the systems may increase people’s confidence in managing their health on the web [57].

To attain an inclusive digital society, web-based health services should meet the particular needs of vulnerable groups. In particular, there should be a wider understanding of specific personal vulnerabilities and how they affect web-based health services [3]. Facilitating digital inclusion among vulnerable groups means helping them develop skills and confidence in using technology [24]. Thus, a conscious, considered, and flexible choice of digital tools and operating environments based on the client's needs is essential in developing an inclusive social and health care service culture [16].

### Strengths and Limitations

This study is based on an extensive national survey, which represents the Finnish population quite well. The respondents of this study were slightly older, more often women, and had a higher education than those seen in the eligible population [45]. The analysis was based on Finnish data, and therefore, the results cannot be readily generalized directly to other countries. Finland is also seen as one of the forerunners in the field of digitalization, with the national digital *Kanta* services performing well and tax-financed universal health care for all residents. However, this study did not examine any national specificities in the Finnish social and health care service system, but rather digital services in general, which, in turn, helps to improve the transferability of the results.

As a final consideration, this study relied on self-reported measures, and consequently, the problems associated with an inflation of the strengths of relationships and common method variance may be an issue here. This problem is decreased because the reliability of the measures we used was quite high. However, we cannot rule out the possibility of residual confounding, despite controlling for many factors, including age, gender, education level, and poverty.

### Conclusions

With the digitalization of public services, the importance of digital inclusion and avoidance of the digital divide is emphasized. This issue concerns not only digital skills and access to web-based services but also people’s attitudes. Thus, supporting the use of web-based services requires both training and influencing people’s ways of thinking. From the perspective of digitalization, individuals with mental health problems are not a cohesive group. Low levels of education, older age, and poverty pose risks of digital exclusion. In terms of general attitudes toward web-based health and social care services, people with mental health problems are no different from those of young people. However, people with mental health problems combined with other risks, such as older age, need special support in using web-based services. According to our findings, women’s digital attitudes were more negative than men’s, especially with regard to their attitude about the importance of face-to-face encounters. On the other hand, among people with poor mental health, men’s digital attitudes are more negative than women´s. Thus, it seems that among people with mental health problems, men seem to be more at risk of digital exclusion.

In addition to social and digital support, attention must be paid to social and economic living conditions. In addition, the development of more accessible, user-friendly, and reliable web-based health care and social welfare services can influence people’s attitudes toward web-based services, thereby preventing digital and social exclusion. Services that combine digital and face-to-face interactions also play a key role in creating challenges for the competence of workers in various areas of health care and social welfare services. In particular, the results of this study emphasize the importance of guidance and counseling.

## Appendix

Multimedia Appendix 1 Factor analysis to determine the dimensionality of attitudes toward web-based health and social care services.

## Abbreviations

MHI: Mental Health Inventory
THL: Finnish Institute for Health and Welfare
