# Supplementary material for: The Associations Between Mental Health Problems and Attitudes Toward Web-Based Health and Social Care Services: Evidence From a Finnish Population-Based Study
Source: J Med Internet Res. 2021 Sep 21;23(9):e28066. doi: 10.2196/28066 (PMC8493458; doi:10.2196/28066)
Supplement: Multimedia Appendix 1 [file jmir_v23i9e28066_app1.docx]

## **Supplementary material**

## **Factor analysis to determine the dimensionality of attitudes toward online health and social care services**

The questionnaire included 11 questions about limiting attitudes (i.e., barriers) that make it impossible or difficult for the respondent to use the online health and social care services. Parallel analysis (Horn, 1965) was used to determine the dimensionality of these items. According to the analysis, a maximum of three factors could be extracted. We conducted an exploratory factor analysis extracting three factors, using robust maximum likelihood, oblique geomin rotation, and sampling weights in Mplus version 7 (Muthén & Muthén, 2012). One item with significant cross-loadings on all three factors was removed (“Electronic services make it slower to access social or health services and delay the care or service process”) to achieve the final factor solution (see Supplementary Table 1). Factor scores were saved for further analysis.

**Supplementary Table 1.** Standardised factor loadings of the final factor solution (N=4051)

| **Item** | **Factor 1** | **Factor 2** | **Factor 3** |
| --- | --- | --- | --- |
| Electronic services do not interest me | **0.90** | 0.01 | 0.00 |
| I do not benefit from using the e-services in any way | **0.93** | -0.02 | 0.02 |
| I think the e-services are unnecessary, as I can contact professionals by telephone during their telephone hours | **0.73** | 0.17 | -0.02 |
| I do not think I will receive good care unless I meet the health care professional face to face | 0.28 | **0.58** | 0.02 |
| Face-to-face interactions cannot be replaced by electronic contacts | 0.00 | **0.79** | -0.02 |
| The non-medical parts of my care do not receive sufficient attention unless I meet the caregiver face to face | 0.00 | **0.88** | -0.02 |
| I cannot be sure that errors, such as those in my medication, will be avoided | 0.00 | **0.68** | 0.21 |
| I am concerned about data security when it comes to my personal details | -0.04 | 0.01 | **0.89** |
| I do not trust e-service providers (the possibility of being cheated) | 0.19 | -0.01 | **0.77** |
| I do not trust that my personal information is kept strictly confidential in anonymous contacts | 0.02 | 0.02 | **0.87** |

*Note.* Loadings > .30 are shown in boldface. The correlations between the factors were r=0.58 (between factor 1 and factor 2), r=0.55 (between factor 1 and factor 3) and r=0.64 (between factor 2 and factor 3.)

### **Bivariate associations between study variables**

Supplementary Table 2. Differences in attitudes toward online health and social care services by mental health status, gender, education level, and poverty status

| Attitude | Poor mental health - no | Poor mental health - yes | p-value | Men | Women | p-value | Education- high | Education-average | Education-low | p-value | Poverty - no | Poverty - yes | p-value |
| --- | --- | --- | --- | --- | --- | --- | --- | --- | --- | --- | --- | --- | --- |
| Lack of interest | -0.09 | 0.18 | **<.001** | -0.05 | 0.04 | **.007** | -0.35 | -0.10 | 0.32 | **<.001** | -0.02 | -0.03 | .688 |
| Need for face-to-face encounters | -0.07 | 0.23 | **<.001** | -0.07 | 0.05 | **<.001** | -0.21 | -0.06 | 0.18 | **<.001** | -0.02 | 0.07 | **.037** |
| Concern for safety | -0.08 | 0.25 | **<.001** | -0.06 | 0.05 | **<.001** | -0.25 | -0.05 | 0.21 | **<.001** | -0.04 | 0.11 | **<.001** |

*Note.* P-values for differences between groups. The differences were examined using t-tests (mental health status, gender, poverty status) or ANOVA (education level). The attitudes were standardised with a mean of zero and a standard deviation of one.


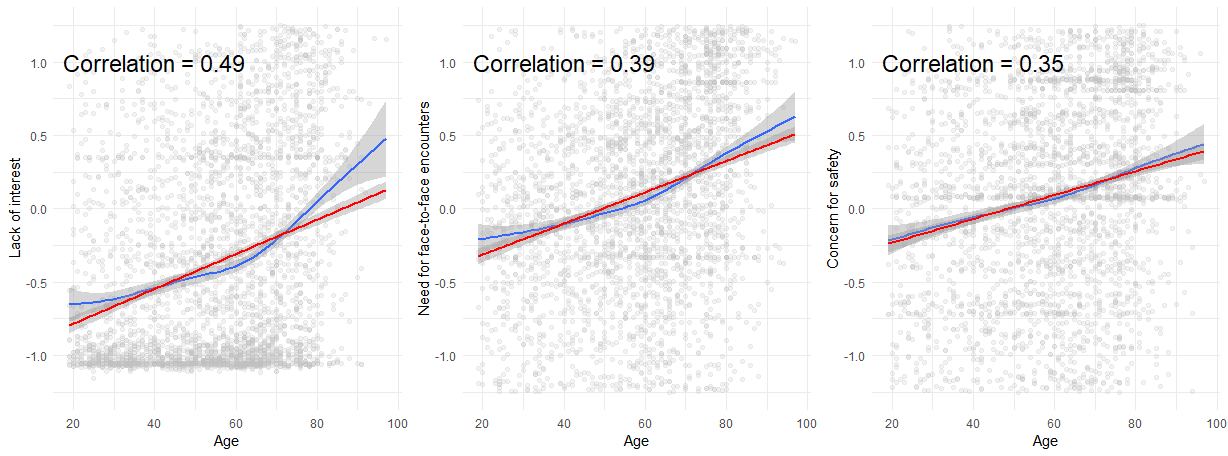


Supplementary Figure 1. The association between age and attitudes toward online health and social care services. The straight red line is the ordinary least squares regression line and the blue curved line is less smooth.

### **References**

### Horn JL. A rationale and test for the number of factors in factor analysis. Psychometrika 1965; 30(2):179–185. doi:10.1007/BF02289447

Muthén LK, Muthén BO. Mplus user’s guide. Los Angeles, CA; 2012.
